# On the measurability of consciousness

**DOI:** 10.3389/fpsyg.2025.1470722

**Published:** 2025-05-30

**Authors:** Satya Pradhan

**Affiliations:** Center for Vedantic Studies, Hayward, CA, United States

**Keywords:** consciousness, measurement problem of consciousness (MPC), consciousness measurement system (CMS), problem space analysis, process workflow, MPC use cases

## Abstract

With the recent advances in neuroscience and brain scanning technologies, there is an increased interest in the measurement problem of consciousness (MPC). The development of a consciousness measurement system (CMS) is still in its infancy without a formal framework and established design approach. This article presents a novel consciousness measurement framework based on consciousness theories and engineering concepts such as measurement workflow, problem space analysis, and observability. The framework proposes measurability criteria, applies them to different use cases, and identifies whether existing theories and technologies can measure consciousness attributes for specific use cases. Researchers and engineers can use this framework to determine the feasibility of CMS for individual use cases.

## 1 Introduction

The nature of consciousness is an entirely private affair. The experience is exclusive to the individual, and no one else has any access to this experience except witnessed external behavior, including what the individual chooses to report verbally or through other means (Koch, 2019). With recent advances in large language models (LLM), artificial intelligence (AI), and quantum computers, machines are starting to simulate human-like behavior, such as intelligence and creativity. It may not be far into the future when machines exhibit behaviors resembling human emotions and feelings. With the adoption of these technologies, a frequent question is if these AI systems are actually conscious or just simulating conscious behaviors. What about animals? Which animals have consciousness, and which ones do not? How do we know if coma victims or patients in a vegetative state are conscious? How can a family know when a patient in a minimally conscious state is truly conscious?

The fundamental question is how to determine if a person, an animal, or anything is actually conscious and not just simulating various aspects of consciousness. This question is not new. It has been around in some form for ages, yet neither philosophy nor science has been able to provide acceptable answers until recently. The measurement problem of consciousness (MPC) is about measuring the subjective nature of conscious experience (a.k.a. phenomenal consciousness), which is inaccessible to direct measurement and relies on indirect observation or reports. Sandberg et al. (2010) studied subjective awareness using different measures, such as the perceptual awareness scale (PAS), confidence ratings (CR), and post-decision wagering (PDW). Gamez (2014) proposed a framework of definitions and assumptions to explain how consciousness can be measured. This addresses the problems associated with first-person reports and avoids the issues with the causal closure of the physical world. In another study, Jimenez et al. (2024) discussed several approaches to measuring consciousness, such as objective (i.e., performance-based) and subjective (i.e., report-based) measures of awareness. Browning and Veit (2020) looked into 3 distinct yet closely related problems relating to the MPC: (i) the indicator validity problem, (ii) the extrapolation problem, and (iii) the moral problem. Since the subjective nature of consciousness can only be measured through first-person reports, researchers must address the problems associated with the accuracy of first-person reports and the possibility of non-reportable consciousness.

Researchers have also developed different measurement technologies such as neural complexity, information integration theory (IIT), and correlates of consciousness. Seth et al. (2006) have formally analyzed 3 quantitative techniques to measure human consciousness based on dynamical complexity in the neural systems underlying consciousness. The techniques include neural complexity, information integration, and causal density. They found that no single measure fully captures the multidimensional complexity of conscious neural systems and conclude that a satisfactory measure is likely to be one that combines both qualitative and quantitative elements. Kim et al. (2018) used measurements from a multichannel electroencephalogram (EEG) to estimate consciousness in the human brain using Tononi’s (2008) IIT. The study represents a new practical approach to the application of IIT, which may be used to characterize various physiological (sleep), pharmacological (anesthesia), and pathological (coma) states of consciousness in the human brain. The IIT has also been used by Arsiwalla and Verschure (2018) to measure the complexity of consciousness in humans. The goal of this study was to create a lookup table of measures of consciousness with particular emphasis on clinical applicability. In another study, Hunt et al. (2022) examined several categories of tests for making reasonable inferences about the presence and complexity of consciousness. They labeled them as the measurable correlates of consciousness (MCC), which includes 3 subcategories: (a) neural correlates of consciousness (NCC), (b) behavioral correlates of consciousness (BCC), and (c) creative correlates of consciousness (CCC).

Even with all the progress, the development of consciousness measurement systems (CMS) is still in its infancy and does not have a formal measurement framework and established design approach. To the best of our knowledge, no research in open literature addresses consciousness measurement from a product development perspective, leaving many basic design and engineering feasibility questions unanswered. The CMS needs to be designed for different scenarios or use cases. The existing theories and technologies may be sufficient to measure consciousness in some use cases, though they may not be adequate for other scenarios. In this article we use product design principles (e.g., measurement process workflow and problem space analysis) to define a novel framework for analyzing the measurability of consciousness. Through this framework we propose measurability criteria, apply them to different use cases, and identify whether existing theories and technologies can measure the required attribute of consciousness in a use case. Researchers and engineers can use this framework to study the feasibility of CMS for specific use cases.

The paper is organized as follows. In Section 2, we describe the conceptual framework for measuring consciousness including the measurability criteria. In the next section, we provide details of consciousness measurement workflow. The theories and technologies available for designing different components of this workflow are also presented in this section. In Section 4, we apply the measurability criteria to 3 broad categories of use cases. This is followed by a summary of this study in the last section.

## 2 Framework for consciousness measurement

Increased focus on MPC has resulted in a new wave of interest in the philosophical foundations of measurement that address basic questions such as what measurement actually is, what conditions have to be fulfilled for a process to be accepted as a measurement (a.k.a. measurability criteria), and what are the basic assumptions required for a measured quantity to be meaningful (Mari et al., 2021). In this section we introduce a novel framework to define measurability criteria for different MPC use cases. In the framework we use concepts from product design (e.g., process workflow, problem space analysis, and the idea of observability from system theory) to identify different use cases and define measurability criteria for these use cases.

### 2.1 CMS use cases

As compared to other measurement systems, the CMS has a unique challenge in terms of what exactly is being measured. There is no widely agreed definition of consciousness (Searle, 2000). The following is a list of common measurement questions that researchers attempt to answer (Browning and Veit, 2020; Hunt et al., 2022; Seth et al., 2008).

- How do we determine the presence and properties of consciousness?
- How can we know if any person, animal, machine, AI, or anything is conscious and not just simulating various aspects of consciousness?
- Are AIs actually conscious or just simulating conscious behavior?
- How do we know if coma victims or patients in vegetative states are conscious?
- How can a family know when a patient in a minimally conscious state is truly conscious?
- Do animals have consciousness? Which animals have consciousness, and which ones do not?
- How do we measure the level of pain and suffering in animals involved in animal agriculture and animal experimentation?
- How can we measure whether and to what extent a particular motor, sensory, or cognitive event is consciously experienced?

Many of these scenarios are completely different from each other and cannot be measured by the same consciousness measurement system. For example, it is very likely that measuring whether a coma patient is conscious and whether a humanoid robot is conscious will require completely different measurement systems. None of the studies in open literature addresses scenario-specific measurement. This is primarily because the development of CMS is still in its infancy and does not have a formal measurement framework and an established design approach.

To design a CMS specific to individual scenarios, researchers could use a formal product design approach that is typically used for solving engineering problems. This involves a sequence of steps (Goel and Pirolli, 1992):

- Exploration and decomposition of the problem (i.e., analysis);
- Identification of the interconnections among the components;
- The solution of the subproblems in isolation;
- The combination of the partial solutions into the problem solution (i.e., synthesis).

**Figure 1:**
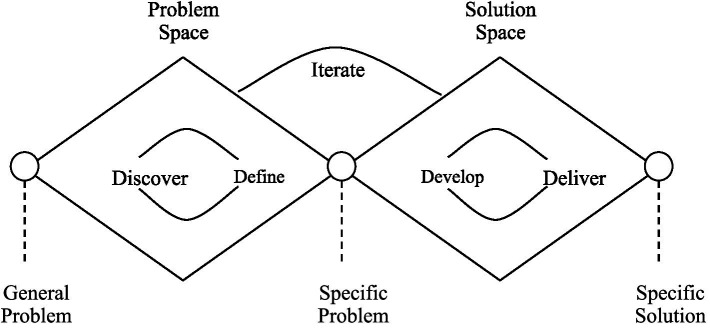
Problem space and solution space in product design.

In this formal problem-solving approach, the first step is called the problem-space analysis and performed prior to the remaining steps to gain additional insights into the nature of the problem itself. The *problem space* is the problem and everything associated with the problem, including the stakeholders, history, and philosophy of the problem. The stakeholders include those who contribute to the problem, those who benefit from the problem, and those who feel the problem most deeply as pain (Maedche et al., 2019). The *solution space*, in contrast, constitutes the products, services, and policies that help address a particular problem. Defining *the problem space* and writing a problem definition are the first steps to solving a problem. In other words, “what” the product needs to accomplish for users or customers is the *problem space* and “how” the product would accomplish it is the *solution space*. A schematic representation of the problem and solution space is given in Figure 1.

By carefully reviewing the questions that a CMS needs to answer, we can divide the CMS problem space into 3 broad categories or use cases. Each use case consists of a set of questions that may require distinct technology to solve.

- *Potentiality of consciousness* – this use case addresses questions about whether the object of measurement has the potential to be conscious. The objects of consciousness include every possible system, including humans, AI systems, animals, biological organisms, silicon-based machines, and extra-terrestrial autonomous systems (if and when we find them).
- *Presence of consciousness* – once we know or assume that the object of measurement is capable of having consciousness, the next question is whether the object is actually conscious during measurement. This measurement can be used to find if a coma patient or a person after a traumatic injury is actually conscious.
- *Degree of consciousness* – if the object of measurement is conscious, the question is to find the degree of consciousness. Note that the previous use case for the presence of consciousness is a boundary condition of this use case, which covers the following questions.
  ○ How do we know if coma victims or patients in vegetative states are conscious?
  ○ How can a family know when a patient in a minimally conscious state is truly conscious?
  ○ Can we find the type of consciousness (e.g., coma, vegetative state, and sleep phase) of a patient after an accident?
  ○ What is the degree of consciousness of a monkey as compared to humans?
  ○ Do dolphins have consciousness? If yes, how does it differ from human consciousness?
  ○ Does a bird have the same level of consciousness as a dolphin?

The use case for the presence of consciousness is a subset of the use case for the degree of consciousness. For example, if we have an instrument to measure the degree of consciousness on a scale of 0–10, a zero value can represent the absence of consciousness, and any nonzero value between 1 and 10 can represent the presence of consciousness. However, given the primitive state of technology for measuring consciousness, it may be worthwhile to design a measurement system that can give a binary output (present or absent) for the presence of consciousness. Note that this paper presents a simple problem space analysis to demonstrate the application of the measurability criteria. The actual design of a CMS may include many other scenarios, such as phenomenal and access consciousness (Block, 1997), different neurological conditions (e.g., synesthesia, phantom limb syndrome, and Capgras syndrome) (Ramachandran, 2004), and animal behavior (Safina, 2016).

### 2.2 Measurement workflow

**Figure 2:**
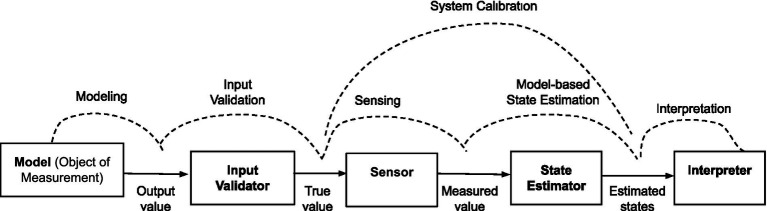
Components, signals, and functions in measurement process workflow.

The measurement process workflow consists of several components that interact with each other through the exchange of information (a.k.a. signals) to perform specific functions. Depending on the task, the workflow can be broken down into constituent parts in different ways. For example, the workflow breakdown by Bentley (2005) consists of input sensing, signal conditioning, signal processing, and data presentation to produce output signals of sensors. This workflow breakdown is helpful for the design of sensors for physical systems. We propose a more general workflow breakdown (Figure 2) for end-to-end system-level analysis of the measurement system where sensing is considered as one of the sub-systems.

The act of measurement presupposes the existence of a component called the “object of measurement,” having states or attributes of interest. These internal states may or may not be measurable. The object of measurement produces specific signals or outputs (a.k.a. measurands) that are functions of the internal states and can be measured. The relationship between the outputs and the states is established by a mathematical function defined as part of modeling. The outputs of the object of measurement can be fed as input into another component called the “sensor” that measures the measurands. The sensor measures any signal that is provided as an input. It does not distinguish between scenarios where the sensor input comes from the real “object of measurement” or some simulated system. Therefore, we need to ensure that input to the sensor is truly the “measurand” that we want to measure. This function is performed by a component called “input validator.” The input validation is typically performed by a conscious agent (i.e., human) or guaranteed during system design by ensuring that the sensor is appropriately connected to the object of measurement. However, input validation cannot be guaranteed in all measurement scenarios, particularly when humans are not involved, and sensors are not explicitly connected to the object of measurement. Therefore, we have “input validation” as a separate component between the object of measurement and the sensor.

The input to the “sensor” is the “true value,” which is the output of the “input validator.” The output of the sensor is the measurement of the “true value” and is called the “measured value.” The “measured value” can then be processed by another component called a “state estimator” to calculate the values of the states or attributes of the object of measurement. The estimated states are then interpreted by the “interpreter,” the last component in the measurement workflow. In addition to these functions, the measurement system must be calibrated against a reference system for the interpreter to make sense of the estimated states. This process is typically done during design validation before the measurement system is used in real life. The schematic diagram of all components, signals, and processes in the measurement workflow is shown in Figure 2.

**Figure 3:**
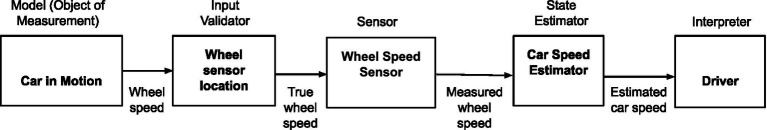
Measurement process workflow of a car speed measurement system (speedometer).

An example of the measurement process workflow for a speedometer is shown in Figure 3. The goal of the measurement process is to measure the speed of the car. The speedometer cannot directly measure the car speed. Instead, it measures the rotational speed of the wheel and then estimates the car speed. In this case, the moving car is the object of measurement. The car speed is the state of the system that we want to measure. The wheel speed is the output of the object of measurement. The system model or the relationship between the output and using the state estimator that is derived from the mathematical formula in Equation 1.


(1) $$W=\frac{S}{D\times \pi \times \mathrm{CF}}$$

where W is the wheel speed, S is the car speed, D is the diameter of the car tire, *π* is the mathematical constant (π = 3.14) representing the ratio between the tire’s perimeter and diameter, and CF is a constant factor for unit conversion. The wheel speed (W) is measured by a speedometer sensor, also known as a wheel speed sensor (WSS), which is a device based on magnetic or semiconductor technologies. The input validation is done by placing the sensor at the right position on the wheel shaft of the car. This is done during the manufacturing process. Once we know the wheel speed (W), the car speed (S) can be estimated using the state estimator that is represented by the mathematical formula.


(2) S=W×D×π×CF

The values for D and CF are known for each car type. The measurement system calibration for the speedometer is done during car manufacturing. The car speed is finally shown in the speedometer dial or digital display and can be used by the driver. In this case, the driver is the interpreter of the estimated car speed.

### 2.3 Measurability criteria

A measurement process is meaningful only if the interpreter can interpret the estimated value to make sense of the states of the object of measurement. In other words, the states are called “measurable” (i.e., can be measured) only if there is a measurement workflow where the interpreter can interpret the “measured value” to make sense of the “states.” This can be achieved only if each of the components in the workflow (Figure 2) performs its own function accurately. The set of criteria for accurate functioning of each component is called “measurability criteria” and can be defined as follows.

a. *Ability to model* – the states or attributes that describe the behavior of a system may not always come out of the system as output and, therefore, may not be directly measured by a sensor. Therefore, we need a mathematical model to define the output in terms of system states. This model is essential to reconstruct or estimate system states using the measured value. To put it differently, we cannot estimate system states and a measurement system cannot be designed without a model.
b. *Input validability* – the sensing system measures any acceptable signal that is provided as an input. It does not have any way to know whether the input is coming from the real object of measurement or from some simulated object. Therefore, something or somebody outside the sensing system needs to validate the source of the input signal. The “input validation” may sound trivial in most real-life scenarios where a conscious agent performs the measurement. However, it is an essential function of the measurement workflow that needs to be implemented for autonomous systems. For the overall measurement process to be effective, we must be able to validate that the input to the sensor is the output of the actual object of measurement.
c. *Ability to sense* – for a measurement system to be effective, it must have the “ability to sense” the output signal (a.k.a. measurand) of the object of measurement. The output of the sensor system is the most essential information in the measurement workflow and is used by the state estimator to reconstruct the system states.
d. *Observability* – the state estimator should be able to estimate the states or attributes using sensor outputs. This is called “observability” in system theory (Kalman, 1960). The simplest observable system is where the system output (i.e., measurand) is the same as the system state (e.g., measuring the height of a person). The speedometer in a car (Figure 3) and a 3-dimensional attitude estimator of a spacecraft are examples of more complex state estimators. The speedometer uses the measurement of the wheel’s rotational speed to estimate the car’s linear speed. In the spacecraft example, the 3-dimensional attitude of a spacecraft is estimated using the measurement of the earth’s magnetic field (Psiaki et al., 1990). The observability of complex systems depends on the mathematical model used to describe the system output in terms of its states.
e. *Interpretability* – the estimated states must be “interpretable” by the interpreter so that one can make sense of the measured values in terms of the states of the object of measurement.
f. *System calibratability* – we make sense of measurement only by comparing it with a previously established reference. This requires the availability of a reference and the ability to correlate the measurement with the reference. The ability to calibrate the system (a.k.a. system calibratability) is essential for an effective measurement.

## 3 Consciousness measurement workflow

The consciousness measurement workflow has exactly the same components as a generic measurement workflow presented in Figure 2. But theories and technologies used to implement each component depends on what is being measured. In this section we provide a summary of theories and technologies currently available for different components of the consciousness measurement workflow. A detailed review of these theories is outside the scope of this study. However, enough details are presented to facilitate discussion on the measurability of CMS use cases in Section 4.

### 3.1 Model

A scientific model of the object of measurement is the essence of a measurement process. No measurement is possible without this model. Different theories are available in open literature to model consciousness. In a recent review paper, Sattin et al. (2021) assessed 1,130 consciousness-related articles published between 2007 and 2017, analyzed 68 selected articles, and identified 29 theories of consciousness. Review of these models is outside the scope of this paper. However, a few of the major theories relevant to the measurement of consciousness are presented in this section. Materialistic worldview has been the basis of several theories of consciousness, such as worldly discrimination theory (Eriksen, 1960), integration theory (Edelman, 2003; Tononi, 2008), and higher-order thought theories (Seth et al., 2008). This worldview proposes that phenomenal subjectivity (feeling and experience) is a consequence of neuronal activity in the brain. It is assumed that consciousness emerges from the underlying biological processes in the brain and can be represented by the complexity of conscious neural systems (Seth et al., 2006). There is no explanation of how the underlying neurophysiological processes in the brain give rise to consciousness. This is called the hard problem of consciousness (Chalmers, 1995).

These theories give useful models to measure the degree or complexity of consciousness of human subjects (e.g., persons in a coma or vegetative state), who are assumed to be conscious under normal conditions. However, if we are trying to know if a system (e.g., a humanoid robot) has the capability or potential to be conscious, assuming that the system is capable or not capable of being conscious becomes a circular argument. Other theories, such as panpsychism (Chalmers, 2016) and cosmopsychism (Keppler and Shani, 2020), attempt to overcome the aforementioned limitation by considering the mind or a mindlike aspect as a fundamental and ubiquitous feature of reality. However, there is no specific mathematical model showing how the atomic consciousness of panpsychism is combined and the ubiquitous field of consciousness of cosmopsychism is divided to form complex human consciousness. Without such details, one cannot use these theories to develop a model for consciousness measurement.

There are other theories of consciousness such as the yogic theory of consciousness (Tripathi and Bharadwaj, 2021) and advaitic theory of consciousness (Ganeri and Shani, 2022; Vaidya and Bilimoria, 2015) that include the concept of “experiencer” based on metaphysical concepts from Hindu philosophy. Like panpsychism and cosmopsychism, these theories do not provide a mathematical model to represent experience and feeling associated with consciousness.

### 3.2 Input validator

The complexity of input validation depends on how the measurement is performed. It may be a trivial task when a conscious agent (e.g., human) performs the measurement. However, it may not be so apparent for use cases where the measurement is done by autonomous systems (e.g., humanoid robots). The input validation may be a simple activity like a visual inspection to ensure the sensor is placed on the actual object of measurement, or it may require a complex process to validate that the input signal is coming from the actual object of measurement. The details of input validation vary from case to case and need to be designed for individual use cases using existing technology.

### 3.3 Sensor

There are 3 broad categories of sensing technology currently available for consciousness measurement: behavioral, neurophysiological, and computational. Before the advent of brain scanning technology, the use of measurable behaviors was the only methodology available to measure and understand consciousness. In fact, the world’s first recorded map of consciousness in Vedantic literature from the 7th century BCE or earlier used 3 observable states of consciousness–the waking state, the dreaming state, and the state of dreamless sleep. Later texts added a 4th state called “Turiya” or the state of pure awareness (Thompson, 2015). Behaviorism has been widely used in the study of psychology and consciousness in the 19th and the 20th centuries (Lashley, 1923). More recently, behavioral measures and associated consciousness reports have been used to calibrate neurological brain measures (Gamez, 2014; Seth et al., 2008).

In the category of neurophysiological measurement, the NCC have received the most scientific attention as a means for measuring the complexity of consciousness. The term was first coined by Crick and Koch (1990) to determine what parts of the brain are necessary and sufficient for conscious experience. The NCC are defined as the minimal neuronal mechanisms jointly sufficient for any one specific conscious percept. They have been used to develop tests for consciousness in patients using various neuroimaging tools such as EEG, magnetoencephalography (MEG), functional MRI (fMRI), and transcranial magnetic stimulation (TMS) (Dehaene, 2014). A measurement framework using NCCs, along with the BCCs and CCCs, has been proposed by Hunt et al. (2022) to develop CMS.

The computational model of consciousness is based on the proposition that phenomenal experience is a consequence of neuronal activity in the brain. Neural complexity defines the extent to which a system is dynamically segregated and integrated (Tononi et al., 1994). The neuronal activities can be measured by 3 different models: neural complexity, information integration, and causal density, which explicitly attempt to quantify the balance between integration and differentiation exhibited by a neural system (Seth et al., 2006). The actual measurement of consciousness using integrated information has been reported by Oizumi et al. (2016), and Mediano et al. (2018).

### 3.4 State estimator

The state estimation function uses the sensor output (i.e., measured value) to estimate the states or attributes of the object of measurement. In the simple example of a speedometer (Figure 3), the state estimation function estimates the car speed using the wheel speed measurement from the sensor (Equation 2). For complex dynamic systems, the state estimation function needs to use a multidimensional time-dependent (i.e., dynamic) mathematical model. The validity of the state estimator in these dynamic systems is measured by mathematical conditions called “observability criteria” (Kalman, 1960). In the context of CMS, we do not have any theory that gives a mathematical model for subjectivity of consciousness. Most of the available theories provide models for measuring complexity of consciousness. Therefore, the validity of the state estimator will depend on the use case and needs to be defined using the mathematical model available for that use case.

### 3.5 Interpreter

Interpretation is the last step in any measurement workflow and is typically done by a conscious agent (i.e., a human being) or an autonomous system (i.e., a computer). Sometimes, both humans and computers may be engaged in interpretation at different levels in a complex measurement process. In the speedometer example, the human driver is the final interpreter of the car’s speed. At the same time, the onboard computer is the interpreter for measuring wheel speed, which is used to calculate the car speed. The goal of the interpretation process is to understand the measurement for the specific use case and make it worthwhile for the end user. The design of the CMS needs to explicitly address the details of how interpretation needs to be done. The details of interpretation depend on the use case and available technologies.

### 3.6 System calibration

Every measurement system must be calibrated to establish the accuracy of the device. In measurement technology, calibration is the comparison of measurement values delivered by a device with those of a calibration standard of known accuracy. The calibration standard for engineering systems is typically defined by a national or international standard. Like other measurement systems, the CMS should also be calibrated using a standard before it is used in real-life applications. There is not much work done in the area of CMS calibration standards. Gamez (2014) proposed to create a standard for calibration using verbal and other behavioral reports from human agents, who are assumed to be conscious. This standard can be used to develop CMS for use cases where the object of measurement can have consciousness. There is no measurement standard currently available for the potentiality of consciousness. The calibration standard for CMS is in a very early development stage and will require refinements over time.

## 4 Application of measurability criteria to CMS use cases

As discussed in Section 3, many technologies are currently available to implement different functionalities of the consciousness measurement workflow. Given all these technologies, is it possible to develop a CMS? The answer to this question depends on the use case under consideration and whether the available technologies can meet the measurability criteria (Section 2.3) for the use case. In this section, we apply the measurability criteria for the use cases defined in Section 2.1 to identify the feasibility of CMS for these use cases. Note that the use case for a real-life CMS will be more detailed and likely be a subset of the general use cases discussed in this study. However, the methodology to identify the feasibility of a CMS will remain the same. It should be emphasized that consciousness theory and sensing technology are active research areas where new ideas are developed regularly. Therefore, the conclusions of this section may change as new theories and technologies are available.

The design of three out of six components of the CMS workflow (i.e., model, sensor, and system calibration) depends on consciousness theories and technologies. The design of the remaining three components (i.e., input validator, state estimator, and interpreter) can be developed using appropriate technologies available during design. Therefore, the discussions in this section will focus on the measurability criteria for the first three components that depend on consciousness theories and technologies.

### 4.1 Potentiality of consciousness

This use case addresses questions of whether an object of measurement (e.g., animals, AI systems, silicon-based machines, quantum computers, and potentially extra-terrestrial autonomous systems) has the potential to be conscious. Is it possible to measure if these objects are capable of having first person subjective experience (a.k.a. phenomenal consciousness)? In consciousness measurement literature, researchers have attempted to perform subjective measurement of awareness based on reports generated by the object of measurement. According to research by Jimenez et al. (2024), one can measure the feeling of a person based on what the person says about their feeling. This is the report-based measurement of an individual’s experience and measures the behavior of the person. It does not measure whether the object that is producing the report is capable of having subjective experience. For example, a chatbot running on silicon-based computers can produce a report simulating human experience but does not experience the conversation. Measurement of a report of experience is not necessarily the same as measurement of the ability to have experience.

A mathematical model is essential for the development of any measurement system. The model provides a structured measurement framework that translates real-world phenomena into quantifiable terms, allows for precise analysis and interpretation of data by defining relationships between variables (i.e., states and outputs), and enables the calculation of unknown values (i.e., states) based on measured data (i.e., outputs) (Khan and Finkelstein, 2011). The main challenge in measuring potentiality of consciousness is the absence of a mathematical model to define first-person subjective experience. Some of the existing theories of consciousness–such as higher-order thought theories (Seth et al., 2008), panpsychism (Chalmers, 2016), cosmopsychism (Keppler and Shani, 2020), and the advaitic theory of consciousness (Vaidya and Bilimoria, 2015)–provide a conceptual definition (not a mathematical model) of consciousness. The computational model of consciousness–IIT (Tononi, 2008)–provides a mathematical model that can measure the complexity and degree of consciousness. None of the available theories provide a mathematical model for subjective experience. Therefore, we cannot measure the potentiality of consciousness in an object using current theories and technologies.

### 4.2 Presence of consciousness

If we assume that the object of measurement (humans or animals) has consciousness, there are several theories of consciousness based on neurophysiological characteristics of the brain (Seth et al., 2006) or IIT (Tononi, 2008) that can be used to develop a model for consciousness measurement. Different sensors based on neuroimaging tools–such as EEG, MEG, fMRI, and TMS (Dehaene, 2014)–can be used to design sensors for the CMS. There is no widely accepted calibration standard available for calibrating a CMS. However, researchers are starting to propose standards and methods for calibration of CMS for the objects of measurement that are assumed to have consciousness, humans (Gamez, 2014). The calibration standard for CMS is in a very early development stage and has a long way to go. But, there is no conceptual challenge in defining a calibration standard for measuring the presence of consciousness in objects that are capable of being conscious. Developing other components in the measurement workflow (i.e., input validator, state estimator, and interpreter) can be done using available technologies. There is no conceptual issue with the feasibility of these functions. It should be emphasized that developing different components of a CMS is not an easy task and may need many years of effort by numerous engineers. What we are discussing here is the feasibility of a design using existing theories and technologies. All the measurability criteria as defined in Section 2.3 can be met for this use case using existing consciousness theories and technologies.

### 4.3 Degree of consciousness

The use case for the presence of consciousness is a limiting case of the degree of consciousness. Therefore, the feasibility of a device for measuring the degree of consciousness is exactly the same as the presence of consciousness. However, the actual device for measuring the degree of consciousness will most likely have much more complex technology than the device for measuring the presence of consciousness. From a measurability perspective, all the criteria defined in Section 2.3 can be met using existing theories and technologies for the degree of consciousness.

A summary of the measurability of consciousness for different use cases is given in Table 1. The symbol “✖” in the table means that the specific measurability criteria for the use case cannot be met with existing theories and technologies. The symbol “✓” means that the specific measurability criteria for the use case can be met with existing theories and technologies. It should be emphasized that the real-life design of a CMS requires more detailed problem space and measurability analysis. The specific CMS use cases for such real-life designs will most likely be a subset of the general use cases used in this study, and the conclusions for the specific use cases may differ from those for the general use cases. However, the approach to analyzing the problem will remain the same.

**Table 1:** Measurability of consciousness for different use cases.

Measurability criteria	Potentiality of consciousness	Presence of consciousness	Degree of consciousness
Ability to model	✖ There is no mathematical model available for subjectivity.	✓	✓
Input validability	✖ Other measurability criteria cannot be met without a model.	✓	✓
Ability to sense		✓	✓
Observability		✓	✓
Interpretability		✓	✓
System calibratability		✓	✓

## 5 Summary

In this paper, we presented a formal framework to define and analyze the measurability of consciousness using product design principles, such as measurement process workflow and problem-space analysis. The framework defines several CMS functions within the measurement workflow. These are: (i) modeling of consciousness states and outputs, (ii) validation of sensor input signals, (iii) sensing, (iv) state estimation, (v) interpretation, and (vi) system calibration. Each CMS function requires different theories and technologies for its implementation. For example, the sensing function can be based on behavioral, neurophysiological (e.g., EEG, MEG, and fMRI), computational (e.g., IIT) measurements. Separate measurability criteria were defined to validate each CMS function. These are: (i) ability to model, (ii) input validability, (iii) ability to sense, (iv) observability, (v) interpretability, and (vi) system calibratability. A product design technique called “problem space analysis” is used to identify 3 broad categories of CMS use cases: (i) potentiality of consciousness, (ii) presence of consciousness, and (iii) degree of consciousness. The measurability criteria for each CMS function are applied to different use cases. The objective was to identify CMS functions that can or cannot be designed for different use cases using existing theories and technologies. For example, the absence of a mathematical model for subjective experience is an obstacle to designing a CMS for measuring the potentiality of consciousness. However, it should not be a problem for measuring the degree of consciousness.

The key contribution of this paper is a novel consciousness measurement framework that uses measurement workflow, design principles, and consciousness theories. The framework proposes a set of measurability criteria that are applied to different CMS use cases. Researchers and engineers can use this framework to determine the feasibility of CMS for individual use cases.

## Data Availability

The original contributions presented in the study are included in the article/supplementary material, further inquiries can be directed to the corresponding author.
